# Potential use of telephone-based survey for non-communicable disease surveillance in Sri Lanka

**DOI:** 10.1186/s12889-017-4993-1

**Published:** 2017-12-29

**Authors:** H. M. M. Herath, N. P. Weerasinghe, T. P. Weerarathna, A. Hemantha, A. Amarathunga

**Affiliations:** 1 Department of Medicine, Faculty of Medicine, University of Ruhuna, University Unit, Teaching Hospital, Karapitiya, P.O. Box 70, Galle, Sri Lanka; 2 Department of Microbiology, Faculty of Medicine, University of Ruhuna, Galle, Sri Lanka; 3 Department of Medicine, Faculty of Medicine, University of Ruhuna, Galle, Sri Lanka

**Keywords:** Telephone survey, Prevalence, NCDs, Diabetes, Dyslipidaemia

## Abstract

**Background:**

Telephone survey (TS) has been a popular tool for conducting health surveys, particularly in developed countries. However, the feasibility, and reliability of TS are not adequately explored in Sri Lanka. The main aim of this study is to assess the effectiveness of telephone-based survey in estimating the prevalence of common non-communicable diseases (NCDs) in Sri Lanka.

**Methods:**

We carried out an observational cross-sectional study using telephone interview method in Galle district, Sri Lanka. The study participants were selected randomly from the residents living in the households with fixed land telephone lines. The prevalence of the main NCDs was estimated using descriptive statistics.

**Results:**

Overall, 975 telephone numbers belonging to six main areas of Galle district were called, and 48% agreed to participate in the study. Of the non-respondents, 22% actively declined to participate. Data on NCDs were gathered from 1470 individuals. The most common self-reported NCD was hypertension (17.%), followed by diabetes (16.3%) and dyslipidaemia (15.6%). Smoking was exclusively seen in males (7.4%), and regular alcohol use was significantly more common in males (19.2%) than females (0.4%, *P* < .001).

**Conclusions:**

Our study revealed average response rate for telephone based interview in Sri Lankan setting. Overall prevalence of main NCDs in this study showed a comparable prevalence to studies used face to face interview method. This study supports the potential use of telephone-based survey to assess heath related information in Sri Lanka.

## Background

Non-communicable diseases (NCDs) are a group of illness that are not due to infectious process and hence are not transmittable from a patient to a healthy person [1]. NCDs account for most of the deaths worldwide, killing more people each year than all other causes combined [2]. Among NCDs, cardiovascular diseases account for most deaths, or 17.5 million people annually, followed by cancers (8.2 million), respiratory diseases (4 million), and diabetes (1.5 million) [3]. Contrary to the popular opinion, people living in low and middle-income countries are disproportionately affected by NCDs and it is estimated that two of every three deaths due to NCDs occur in these countries [4].

Among low and middle income countries, South Asia is one of the worst affected region with NCDs in the world [5]. The prevalence and the adverse health outcomes of NCDs have risen more rapidly in South Asia than in any other geographical region in the world [6]. Even though, demographic dimension of Sri Lanka is different to the rest of the South Asian countries, factors which influence the burden of NCDs are more or less similar [7, 8]. Like the rest of the other nations, there is ongoing rapid urbanization, which has led to unhealthy eating habits, physical inactivity, high rates of smoking and tobacco use in Sri Lanka [7–9]. Urban population enjoys comparatively better income, but, has more westernized lifestyle with physical inactivity and unhealthy eating habits [10]. People in rural area are mainly involving in agriculture and related occupations and have more physically active lifestyle and healthier eating pattern.

Problems associated with NCDs can be reduced by early diagnosis and proper management [3]. Therefore, disease-surveillance system remains an integral part of the battle against growing menace of NCDs [11]. Such system will provide the vital information on the magnitude of the problem and it will in turn help to implement necessary preventive strategies [11]. Furthermore, regular surveillance system would provide useful information on disease pattern which can be used to educate the general public and political leadership. Unfortunately, disease-surveillance system remains inadequate particularly in resource-scarce countries like Sri Lanka [12].

Periodic surveillance of NCDs was initiated by Ministry of Health, Sri Lanka with the help of World Health organization (WHO) [13]. However, these surveillances are primarily face-to-face household interviews, and they are both costly and time-consuming [13–15]. Alternatively, surveillance using telephone interviews has become increasingly used in health surveys, particularly in the developed countries for screening and monitoring of NCDs (e.g. the CDC Behavioural Risk Factor Surveillance System in the US and the Telephone-based Surveillance of Risk and Protective Factors for Chronic Diseases in Brazil [14, 16]. Compared to face-to-face household interviews, telephone surveys are cheap, faster and required less man power to conduct. Furthermore, telephone surveys can be performed more frequently and hence disease trends can be identified early. In addition, telephone surveys give more freedom to access the respondents, regardless of where they live and what they do [15]. Compared to Face-to-face interviews, telephone surveys are less intrusive, warrant greater anonymity and are more convenient for the respondents [15]. It is also an appealing alternative to surveillance involving face-to-face interviews, especially when the access to certain geographical areas is physically challenging.

Even though, telephone surveys tend to be one of the most cost-effective ways of surveillance of NCDs, the main limiting factor is selection bias based on the availability of telephone lines. In Sri Lanka, around 10% of households (2.6 million) have fixed landlines, where as in Galle district, around 12.5% of households own fixed landlines in 2015.

It is a known fact, that people, who own land telephone connections, are comparatively rich and enjoy better quality of life than those without telephone connection. Therefore, it is likely that socially deprived people will be left out from the survey if telephone interviews are used as the only method of surveillance. In addition, there is significant proportion of individuals in the community with undiagnosed NCDs (e.g. diabetes, hypertension) [1, 17]. All these individuals with undiagnosed NCDs will be left out if telephone surveys are used. Apart from the availability of telephone lines, quality of the survey also depends on many other factors such as individuals’ response rate, respondent’s attitudes and their knowledge. Even with these limitations, telephone surveys may still have the potential to be used as a screening method for NCDs particularly in resource poor settings.

There were no previous studies done in Sri Lanka to assess the feasibility and reliability of telephone surveys. Therefore, the present study was designed with main aims of identifying the feasibility and potential challenges of applying telephone surveys for NCDs screening. In addition, the prevalence of self-reported common NCDs (diabetes mellitus, hypertension, ischemic heart disease etc) was assessed based on telephone interview method in this study.

## Methods

### Sampling procedures

This study was carried out in Galle district, which is the most populated district in Southern province of Sri Lanka. It has a population of 1.06 million, dispersed over an area of 1652 km^2^ and has 32,234 households with fixed landline telephones in 2015. This study was a community based cross sectional descriptive study and the study population consisted of all residents living in households with fixed land telephone line (Sri Lanka Telecom telephones). The list of telephone numbers with the household details was obtained from the Sri Lanka Telecom company. It is the main telecommunications provider in Sri Lanka and is the only company providing fixed landline telephones in Sri Lanka. Inclusion criteria for the study were all residents of 20 years or more living in households with fixed Sri Lanka Telecom landline. Exclusion criteria were the member of the household spending majority of their time residing outside Galle district.

### Sample size

The required sample size was 1100 as determined using the equation *n* = *Z* ^2^ × *p*(1-*p*)*Deff*/(*e* ^2^ × *RR*) from the sample size calculator [18] provided by the World Health Organization (WHO) STEP wise approach to NCDs risk factor surveillance (STEPS). We used level of confidence (*Z*) of 1.96 for a 95% CI, a margin of error (*e*) of 0.05, an estimated prevalence of main NCDs (*p*) of 0.5, a design effect (*Deff*) of 1.5, and an expected response rate (*RR*) of 0.5. A total of 1100 subjects were randomly selected from the list provided by the Sri Lanka Telecom from the main regions of Galle district (Galle, Badegama, Hikaduwa, Ambalangoda, Udugame, Hiniduma, and Habaraduwa) using probability proportional to size (PPS) sampling method. We estimated that the number of household required for this study was 975, assuming around 3 adult individuals live in a household and 50% of household would respond positively to participate this study [19].

### Data collection

An initial questionnaire was drafted based on the step 1 section (demographic Information and behavioural measurements section) of WHO-NCD-STEPS instrument version 2.2. [20]. The translation and cultural adaptation of the WHO-NCD-STEPS instrument followed a standardised protocol provided by the authors of the original instrument [21]. This included: a forward translation (English to Singhala) by a professional, registered translator and a blinded independent back translation (from Singhala to English). Three questions of the original questionnaire was further modified to suit for telephone interview. The translated questionnaire was subjected to face and content validity. Face validity of the questionnaire was ensured independently by two experts dealing with epidemiological research. Subsequent to this, the content validity of the questionnaire was done after a meeting with several others including two physicians, an epidemiologist, a psychologist and two laypersons. Final version of the questionnaire was administered by telephone interview method to five randomly selected individuals to improve the clarity of questions.

Data collection was carried out during the period of February 2017 to April 2017. All interviews were conducted by trained pre-intern medical graduates. The households were called during the time period of 8 am to 4 pm during weekdays and introduced about the research. If there was no response from a household at the initial contact, we made repeated calls up to three times, two-three days apart, at different times. All eligible individuals of the selected households were interviewed using the predesigned questionnaire. Verbal consent to disclose information was obtained from respondents before the interview. All initially contacted households were called repeatedly at their convenient times, till we completed interviewing all eligible individuals in the households. However, details were obtained from a next of kin (168/1470) when an eligible individual unable to participate for interview at all occasions of repeated calls or if an individual was too frail or sick to participate. The participants were given opportunity to ask questions and clarify doubts. Furthermore, they were given the telephone numbers of the investigators to clarify the doubts about the study.

### Operational definitions

Following operational definition were used during data collection.

### Hypertension

Any individuals currently on anti-hypertensive medications, used anti-hypertensive medications in the past or claim to have recorded high blood pressure (SBP ≥140 or DBP ≥90 mmHg) at least on two occasions.

### Diabetes mellitus

Any individuals on medications for diabetes at present or in past, or found to have blood sugar 126 mg/dl or above on two or more occasions.

### Dyslipidemia

Any individuals on statin group of medications for dyslipidaemia at present or in past was defined as having dyslipidemia.

### Ischemic heart disease (IHD)

Any individuals who was treated for IHD at present or in past were considered as having IHD.

### Stroke

Any individuals who had been treated for stroke or transient ischemic attack were considered as having stroke.

### Cancer

Cancer diagnosis was defined by the positive response (Yes) to the question “Have you ever been told by a doctor or other health professional that you had cancer”.

### Asthma

Individuals who responded “yes” for the question “Did a doctor ever tell you that you had asthma?” were classified as having asthma. Respondents who reported not having been told by a doctor that they had asthma were classified as not having had asthma.

### Smoking

Current smokers were defined as individuals who gave a positive response to the following questions. “Do you currently smoke any tobacco products, such as cigarettes, cigars or pipes?” Heaviness of smoking was assessed with the following questions: “On average, how many times a day/week do you smoke?”. “Light smoker (≤10 cigarettes daily), moderate smoker (10- ≤ 20 cigarettes daily), and heavy smoker (>20 cigarettes daily)”.

### Alcohol use

Individuals, taking one or more drinks per day in one or more occasions over last 1 month were considered as alcohol user in this study.

### Salt restriction

Individuals who responded to the following question by saying “rarely” or “never” were considered as restricting salt intake. “How often do you add salt or a salty sauce such as soya sauce to your food right before you eat it or as you are eating it?”

### Data analysis

The data analysis was carried out in relation to each specific objective using SPSS (SPSS Inc., version 11). Participant’s socio-demographic characteristics including age, gender, level of education were reported using descriptive statistics. The prevalence of main NCDs, and measures to achieve a healthy life such as sugar intake, regular exercise, and regular medical check-ups were assessed. The rate of non-responders and the reasons for refusal for interviews were also assessed. The results for categorical variables were presented as proportions with their respective 95% confidence intervals (95% CI). The Chi-square test was used to assess the prevalence of NCDs among gender, and different age groups. Chi-square for trend was used to assess the association between age with diabetes and hypertension. Multiple logistic regression models were constructed relating the behavioural risk factors (tobacco smoking, alcohol consumption, salt and sugar restriction, and regular medical check-ups physical activity as dependent variables which were modelled individually to the demographic variables (predictor) i.e. age groups, and, gender). One-way analysis of variance (ANOVA) was used to compare three or more subgroups of a continuous variable.

### Ethical approval

Ethical approval for this study was obtained from ethical review committee of Faculty of Medicine, University of Ruhuna, Galle. Informed written consent was obtained from all individuals prior to data collection. Participants were informed of their rights to withdraw from the study at any stage.

## Results

**Fig. 1:**
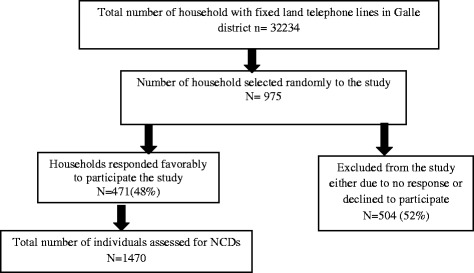
Flowchart showing enrolment process and outcomes

**Fig. 2:**
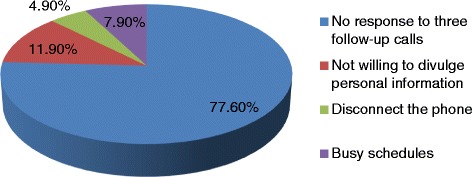
Reasons for “non- response” in the study

Figure 1 shows household recruitment results and the response rate pattern. Out of 975 of households selected for the study, approximately half (471/975) agreed to participate the study, achieving a response rate of 48.3%. Of the households not included for further analysis, majority did not answer (78%) the phone calls made, despite three contact attempts over three different times. Around 12% were not included as they were not willing to divulge their personal or health related information over the phone. Another 5% disconnected the phone without giving any reasons. Remaining 8% declined to participate the study due to their busy schedules (Fig. 2).

For the final analysis, 1470 adults living in 471 households were included. Baseline and demographic characteristics of the study sample are depicted in Table 1. Respondent ages ranged from 20 to 103 years with a mean age of 50.5 years (SD 17.6 yrs.); 31.2% were aged 20– 39 years, 27.6% were 40-59 years and 34.8% were 70 years or older. Males comprised 46.3% of the study sample with a mean age of 50.2 years (SD 17.2 years). Approximately 20% males and females were “unmarried” and there were higher percentage of female widowers (11) compared to male widowers (1).

**Table 1:** Baseline characteristics of the study sample

Gender	*n*	%	mean	SD
Male	684	46.5	50.2	17.3
Female	786	53.5	50.4	17.4
Average earnings/month	n	%		
< Rs.20, 000	231	15.7		
Rs.20,000-50,000	708	48.1		
> Rs. 50, 000	531	36.1		
Highest level of education	n	%		
Less than primary school	24	1.6		
Primary school completed	196	13.3		
Secondary school completed	713	48.5		
High school completed	391	26.6		
University or above	146	9.9		
	Male		Female	
Age category	*n*	%	*n*	%
20-39	212	31.0	246	31.3
40-59	234	34.2	267	34.0
60-79	210	30.8	237	30.2
≥ 80	28	4.0	36	4.6
Civil status				
Single	150	22.2	163	20.7
Married	532	77.5	612	77.9
Widowed	1	.1	11	1.4
Divorced	1	.1	–	–
Ethnicity				
Sinhalese	631	92.2	713	90.3
Sri Lankan Moors	53	7.8	76	9.7

### Prevalence of behavioural risk factors for NCDs

Overall 9.1% of individuals in the study sample consumed alcohol (Table 2). This study showed a lower prevalence of alcohol consumption among women than men (OR: 0.24; 95% CI:(0.12-0.43), and a higher prevalence in those who were in the age group of 40-59 (OR; 3.39; 95% CI:(2.49-3.86) and 60-79 (2.06; 95% CI:(1.92-3.42) compared to those in the age group of 20-39. Among males, alcohol consumption was highest in the age group of 40-59 years. Tobacco smoking in compared to alcohol consumption, was comparatively low with only 3.5% individuals actively smoke. Smoking was exclusively seen in males (7.4%) and was three time higher in the age group of 60-79 years compared to the age group of 20-39 years (OR; 3.01; 95% CI:(2.21-3.91). With regards to the other health promoting behaviours, younger generation (20-39 years) tends to have less health promoting behaviours compared to older individuals. Overall, salt restriction was significantly higher among older individuals (>80 years) in comparison to young individuals (20-39 years) (OR;1.94; 95% CI:(1.27-2.32). Proportion of young individuals (<40 years) having regular medical checks ups was as low as 4.5% compared to 36.4% in the age group of 60-79 years and 41.3% in the age group of 80 or more. Similarly, the practice of dietary sugar restriction was significantly higher in the age groups of 60-79 (OR: 2.07; 95% CI: 1.21-2.78)and ≥80 years (OR; 1.92; 95% CI:1.17-2.56) compared to those in the age group 20-39 years.

**Table 2:** Frequency, percentage and determinants of selected risk factors for NCDs and health-promoting behaviors

	Alcohol use			Tobacco smoking			Salt restriction			Sugar restriction			Regular exercise			Regular medical checkups		
Age category	*n*	%	OR(95% CI)		%	OR(95% CI)	*n*	%	OR(95% CI)	*n*	%	OR(95% CI)	*n*	%	OR(95% CI)	*n*	%	OR(95% CI)
20-39	20	4.4	1	8	1.7	1	71	15.5	1	63	13.8	1	41	9.0	1	19	4.1	1
40-59	69	13.8	3.39* (2.49-3.86)	18	3.6	2.11* (1.87-2.73)	85	17.0	1.12 (0.89-1.23)	117	23.4	1.6 (0.81-1.82)	44	8.8	0.91 (0.56-1.31)	139	27.7	6.24* (2.23-8.91)
60-79	43	9.6	2.06* (1.92-3.42)	23	5.1	3.01* (2.21-3.91)	87	19.4	1.23* (1.04-1.63)	134	29.9	2.07* (1.21-2.78)	43	9.6	1.01 (0.71-1.14)	163	36.4	8.14 (4.87-9.94)
≥ 80	2	3.2	0.93 (0.76-1.08)	2	3.2	1.86 (0.92-2.74)	17	27.0	1.74* (1.27-2.32)	17	27.0	1.92* (1.17-2.56)	7	11.1	1.09 (0.64-1.43)	26	41.3	9.12* (2.34-11.2)
Gender																		
Male	131	19.2	1	51	7.4	1	114	16.1	1	152	22.3	1	100	14.7	1	169	24.8	1
Female	03	0.4	0.24* (0.12-0.43)	0	0.0	0	146	18.1	1.11 (0.92-1.25)	179	22.6	0.91 (0.61-1.23)	35	4.5	0.45* (0.22-0.92)	178	22.6	0.96 (0.61-1.07)
Education																		
Primary or less than school	18	8.1	1	7	3.1	1	31	14.0	1	38	17.2	1	17	7.7	1	51	23.1	1
Secondary school completed	78	10.9	1.21 (0.97-1.34)	27	3.7	1.05 (0.69-1.07)	124	17.3	1.12 (0.97-1.21)	162	22.7	1.23 (0.98-1.32)	62	8.4	1.09 (0.84-1.26)	162	22.7	0.92 (0.61-1.12)
High school completed	28	7.1	0.98 (0.79-1.12)	13	3.3	1.02 (0.65-1.1)	66	16.8	1.19* (1.09-1.32)	94	24.0	1.34* (1.12-1.48)	41	10.4	1.32* (1.13-1.54)	89	22.7	0.91 (0.58-1.14)
University or above	10	6.8	0.84 (0.62-1.06)	4	2.7	0.84 (0.46-0.91)	39	26.7	1.79* (1.23-2.14)	37	25.3	1.42* (1.15-1.54)	15	10.2	1.31* (1.07-1.42)	45	30.8	1.31* (1.02-1.43)
Total	134	9.17		51	3.53		260	17.71		331	22.5		135	9.2		347	23.6	

*Significant Results: *P* value <0.05; OR = Odds Ratio; CI = Confidence Interval

### Self-reported prevalence of main NCDs

Hypertension was the most prevalent self-reported NCD and was found in 17.3% study subjects, followed by diabetes mellitus (16.3%) and dyslipidaemia (15.6%) (Table 3). Cancer and stroke were very rare and seen in less 1% of study population. Hypertension was significantly more common in females (19.7%) than in males (14.6%, *P* < 0.05). Among males, 16.9% were found to have diabetes, as compared to 15.8% among females. Chi-square for trend showed significant association between increasing age with diabetes (χ^2^_trend_-62.11, *P* < 0.001) and hypertension (χ^2^_trend_-72.11, *P* < 0.001) .

**Table 3:** Prevalence of main NCDs according to the age categories and gender

Variables	Diabetes mellitus			Dyslipidemia				IHD		Hypertension			Asthma			Stroke			Cancer		
Age category	*n*	%	*p*	*n*	%	*p*	*N*	%	*p*	*n*	%	*p*	*n*	%	*p*	*n*		*p*	*n*		*p*
20-39	10	2.2	0.014^a^	10	2.2	0.004^a^	1	0.2	0.013^a^	8	1.7	0.001^a^	9	2	0.002^a^	0	0	0.823^a^	0	0	0.069^a^
40-59	82	16.4		65	13		19	3.8		73	14.6		20	4		4	0.8		3	0.6	
60-79	128	28.6		137	30.6		49	10.9		148	33		24	5.4		8	1.8		5	1.1	
≥80	19	30.2		17	27		7	11.1		26	41.3		6	9.5		3	4.8		1	1.6	
Gender																					
Male	115	16.9	0.12+	102	14.9	0.08+	45	6.6	0.04+	100	14.6	0.03+	23	3.2	0.71+	5	0.7	0.13+	2	0.3	0.18+
Female	124	15.8		127	16.2		31	3.9		155	19.7		36	4.6		10	1.3		7	0.9	
Total	239	16.3		229	15.6		76	5.2		255	17.3		59	4		15	1		9	0.6	

^a^One-way analysis of variance (ANOVA) was used + The Chi-square test was used

## Discussion

The main aim of this study is to assess the potential use of telephone survey in epidemiological health research in Sri Lanka. Despite some concerns over the validity of self-reported data collected via telephone surveys, the use of TS in epidemiological research has grown worldwide [6, 12, 16, 17]. As the telecommunication is growing, with its near-ubiquitous penetration and use in most part of Sri Lanka [22], it is interesting to know whether TS can be used for health related research in Sri Lanka. As far as we are aware, this study is the first study done in Sri Lanka to assess the potential use of TS in an epidemiological health study. Notable findings of this study were, (1) there was an average response rate, with nearly 50% agreeing to participate the study; (2) alcohol consumption was common and predominately seen in males particularly in the middle age group; (3) smoking was exclusively seen in males; (3) young individuals were less likely to engage in health promotion behaviours (regular exercise, salt restriction) than older individuals; (4) self-reported prevalence of diabetes and hypertension was comparable to self-reported prevalence observed in face-to-face studies.

Response rate is one of the most important parameter of efficiency and accuracy of telephone surveys [23]. The overall response rate (48%) observed in our study was comparable to the response rates reported in many other health studies. [12, 14, 15, 24, 25] Even though, response rates for telephone surveys can vary depending on many factors, overall responses rates in many countries have been steadily declining for the last two decades. Long established population health surveys such as U.S. Behavioural Risk Factor Surveillance Study (BRFSS) and the University of Michigan’s Survey of Consumer Attitudes reported declining response rate in recent past. [25, 26]. The trend of declining response rate is likely to be due to a confluence factors, such as busy lifestyle, change of attitude, changing patterns of when household residents are at home, and refusals due to confidentiality issues [24]. In our study, only 22% of non-respondents actively declined to participate the study and the majority (78%) did not answer the phone perhaps the household owners were not at home at the time of calls. Due to practical reasons, we were not able to contact non-responders during after-hours. We believe that we would have achieved much higher response rate if the households were contacted during after-hours.

Alcohol use in our study was 9.1% and it was much lower than the reported alcohol use in the previous Sri lankan studies [13, 27]. A study conducted by Katulanda et al. in 2006 showed higher alcohol usage (23.7%) [27]. In this study, alcohol use in males and females was 48% and 1.2% respectively as compared to 19.2% and 0.4% of males and females in our study. Therefore, our study which was carried out roughly 10 years later showed an overall decrease of alcohol use among both males and females. However, a more recent island wide study revealed higher alcohol consumption, with 39.6% of males and 2.4% of females taking alcohol [13]. The apparent difference in alcohol consumption in our study and the previous studies could have been due to socioeconomic status (SES) of the study populations, the criteria used to define alcohol intake and interview methods. As our study included only those who possessed land telephone lines, our study populations was likely to have better SES than the general population Many studies had shown higher alcohol use by people of low SES than those of higher SES [28, 29], the study subjects in our study was likely to be less socially deprived than those in general population in Sri Lanka.

In contrast to alcohol drinking, tobacco smoking was comparatively low, with only 3.5% of individuals were reported to be active smokers. Similar to alcohol use, tobacco smoking observed in this study was also lower than what was reported in recent past [13, 30]. Survey conducted by Ministry of Health, Sri Lanka in 2015 showed overall smoking prevalence of 10.2%, with significantly higher (19.2%) smoking prevalence in males [10]. In comparison, smoking prevalence among males in our study was 7.5%. Apparent difference in smoking observed in our study and the previous studies could have been due to the comparatively affluent population that we studied, however, the observed difference could be due to the effect of telephone based interview method used in our study. Many studies had shown that telephone surveys generally underestimate cigarette smoking rates and therefore, it is possible that the observed smoking rate of 3.1% in our study was lower than the true prevalence of smoking. [31, 32]

Self-reported prevalence of diabetes in our study was 16.3% with no significant gender difference. This figure is alarmingly high considering the fact that true prevalence of diabetes could be 25 to 50% higher than the self-reported prevalence of diabetes [33]. In an island wide survey conducted in 2014 in Sri Lanka showed self-reported diabetes prevalence to be 7.6% which is almost the half of what we found in our study [13]. Our study, being the most recent and comprising more affluent population by virtue of selection based ownership of telephone lines, may explain the differences between the two studies. It is known fact that diabetes is more prevalent in urban areas in Sri Lanka. In 2008, Katulanda et al*.* reported a diabetes prevalence of 16.4% in an island-wide study [34] and in 2013,Pinidiyapathirage et al*.* reported 20% diabetes prevalence in urban setting [35]. Therefore, we assume that the true prevalence of diabetes in our study sample could be as high as 20-22%. As expected, the prevalence of diabetes was higher in older people, with over 28% diabetes prevalence seen in older individuals (over 60 years) as compared to 2.2% in the age group of 20-39 years. The similar rising trend of diabetes with age was reported in many previous studies [36, 37], which perhaps explains the increase in prevalence of diabetes in recent decades.

Hypertension was the most prevalent NCD in our study and was observed in 17.3% of study population with slightly more female preponderance (19.7%). The prevalence of self-reported hypertension in our study was higher than the reported prevalence of hypertension in previous studies [13, 38]. In 2014, an island wide study, involving face to face interview method, showed lower prevalence of self-reported hypertension (6.95%) [13]. However, many other studies had shown significantly higher prevalence of hypertension in Sri Lanka [38, 39]. In 2006, a study conducted in four provinces reported 18.8% and 19.3% of males and females respectively having hypertension [38]. In another community based study involving over 2900 individuals in an urban area showed comparatively higher prevalence of hypertension (30.4%) [40]. In this study, around 30% of hypertensive individuals had previously undetected hypertension [40]. Therefore, it is likely that true prevalence hypertension in our study may be 30% higher than the observed prevalence.

Roughly around 15% of individuals in the study sample either at present or in past were on statin, to be classified as having dyslipidaemia. Unlike diabetes mellitus and hypertension, that were more prevalent in age category of >80 years, dyslipidaemia was more prevalent in age category of 60-79 years. Even though, there were no previous Sri Lankan studies to compare, a large Indian study conducted in a similar setting revealed a similar prevalence of hypercholesterolemia [41]. Furthermore, most patients (>90%) with dyslipidaemia in our study had one or more other NCDs such as diabetes and hypertension and only 8.4% had isolated dyslipidaemia.

This study was conducted primarily to assess the feasibility and potential challenges of using telephone surveys for health related epidemiological study in Sri Lanka. The main strength of our study is the use of a novel, inexpensive, efficient, health-survey methodology that has the potential to improve epidemiological research in Sri Lanka. In addition, inclusion of a random, representative, comparatively large number of adults in the study is also a strength. Despite these strengths, this study has several limitations and therefore, interpretation of the finding of this study should be done in the context of these limitations. Despite we made strenuous efforts to directly interview all adult members in the households, data related to some individuals (around 10%) were obtained from next of kin. Therefore, some of the information (such as tobacco smoking and alcohol use) obtained from next of kin may not be 100% accurate, and hence, can potentially underestimate the true prevalence of these factors. Another important, but unavoidable limitation is the use of self-reported telephone-based information to assess the prevalence of all NCDs. Many studies have shown that the true prevalence of NCDs such diabetes mellitus, dyslipidaemia and hypertension can be significantly higher than the self-reported prevalence of NCDs [42, 43]. Another potential limitation of this study is the sampling bias. By virtue of selecting individuals based on the ownership of land telephones can lead to selecting more affluent individuals to the study and excluding socially deprived individuals. Even with these limitations, our findings support the potential use of telephone surveys in health related research and use of self-reported information for assessment NCDs in Sri Lanka. Since the NCDs are the leading cause of morbidity and mortality in Sri Lanka at present, the pattern and trends generated, along with other indicators, will be useful to evaluate NCDs and other health related parameters in Sri Lanka.

## Conclusion

In conclusion, this study has shown an acceptable and average response rate for telephone based interview in Sri Lankan setting. Overall, prevalence of main NCDs obtained in this telephone based survey showed comparable prevalence, and trends to studies used face-to-face interview method and hence can support the use of telephone-based information to monitor the prevalence and the trends of main NCDs in Sri Lanka.

## Abbreviations

DB: Diastolic blood pressure
IHD: Ischemic heart disease
NCD: Non communicable disease
SBP: Systolic blood pressure
TS: Telephone survey
WHO: World Health organization
